# Systematic review and bibliometric analysis of African anesthesia and critical care medicine research part I: hierarchy of evidence and scholarly productivity

**DOI:** 10.1186/s12871-020-01167-8

**Published:** 2020-09-28

**Authors:** Ulrick Sidney Kanmounye, Joel Noutakdie Tochie, Aimé Mbonda, Cynthia Kévine Wafo, Leonid Daya, Thompson Hope Atem, Arsène Daniel Nyalundja, Daniel Cheryl Eyaman

**Affiliations:** 1 Department of Research, Association of Future African Neurosurgeons, Kinshasa, Democratic Republic of Congo; 2 Department of Neurosurgery, Faculty of Medicine, Bel Campus University of Technology, Kinshasa, Democratic Republic of Congo; 3 Department of Anesthesiology and Critical Care Medicine, Faculty of Medicine and Biomedical Sciences, University of Yaounde I, Yaounde, Cameroon; 4 Human Research Education and Networking, Yaounde, Cameroon; 5 Faculty of Medicine and Biomedical Sciences, University of Yaounde I, Yaounde, Cameroon; 6 Surgery Unit, District Hospital of Batouri, Batouri, Cameroon; 7 Department of Research, International Student Surgical Network, Yaounde, Cameroon; 8 Department of Internal Medicine, Faculty of Medicine, Bel Campus University of Technology, Kinshasa, Democratic Republic of Congo; 9 Faculty of Medicine, Catholic University of Bukavu, Bukavu, Democratic Republic of Congo

**Keywords:** Africa, Anesthesia, Bibliometrics, Global anesthesia, Research

## Abstract

**Background:**

Research is an essential component of Anesthesia, and the contributions of researchers and institutions can be appreciated from the analysis of scholarly outputs. Such analyses help identify major contributors and trends in publication. Little is known about the state of Anesthesia and Critical Care Medicine (A.C.C.M.) research in Africa. We aimed to describe African A.C.C.M. research’s current landscape by determining its productivity per country and point towards possible ideas for improvement.

**Methods:**

The authors searched PubMed, Embase, Web of Science, and Cumulative Index to Nursing and Allied Health Literature (CINAHL) from inception to May 4, 2020, for articles on or about A.C.C.M. in Africa. Studies were selected based on their titles and abstracts. Rayyan software was later on used for data management in the review selection process. Then, the full-text of eligible articles were screened. Data were extracted, and the number of articles per physician anesthesia providers and provider density were calculated. Kruskal Wallis test and Spearman’s correlation were used, and a *P*-value < 0.05 was considered statistically significant.

**Results:**

Of the 4690 articles, only 886 (18.9%) were included in the analysis. The articles were published between 1946 and 2020 in 278 target journals. 55 (6.2%) articles were published in the South African Journal of Surgery, 51 (5.8%) in Anesthesia and Analgesia, and 46 (5.2%) in Anaesthesia. 291 (32.8%) studies were cross-sectional. 195 (22.0%) first authors were from Nigeria, 118 (13.3%) from South Africa, and 88 (9.9%) from the U.S.A. Malawi (1.67), Togo (1.06), and Sierra Leone (1.00) had the highest number of articles per provider. Whereas Ethiopia (580.00), Nigeria (336.21), and Malawi (333.33) had the highest number of articles per provider density.

**Conclusion:**

We identified the most and least productive African countries in A.C.C.M. research and a low-quality hierarchy of evidence in these publications. Hence, the study’s findings may aid in driving the A.C.C.M. research agenda and capacity building in Africa.

## Background

African anesthesia and critical care medicine (A.C.C.M.) face numerous challenges: delayed patient presentations, lack of equipment, lack of an adequate specialist workforce, poor information management infrastructure, and lack of funding [1–3]. These barriers hamper the development of A.C.C.M. practice, education, and research. Out of the three components of A.C.C.M., research has the greatest potential to change the status quo. First, research can help identify the barriers of universal A.C.C.M. access in Africa [4]. Research has been used, for example, to map the workforce deficit and to identify obstacles to the development of A.C.C.M. services in Africa [5, 6]. Also, research can help design, monitor, and evaluate context-specific solutions to previously identified problems [4]. This has been the case with the World Health Organization surgical safety checklist, the LifeBox pulse oximeter, and oxygen provision in low-resource settings [3, 7, 8].

Research can equally improve the career trajectory of individuals and the academic standing of institutions. Their colleagues respect high academic performers, get promoted, and be recruited to more prestigious institutions [9–11]. Similarly, prolific academic institutions attract talented researchers, secure more funding, and gain recognition from the research community and the public [12].

We aimed to describe the landscape of AACM research in Africa, and we hypothesized that the most significant contributions to African A.C.C.M. were from South Africa and Western countries.

## Methods

### Search strategy

A protocol was developed and can be accessed online (10.13140/RG.2.2.28999.32167). PubMed, Embase, Web of Science, and Cumulative Index to Nursing and Allied Health Literature (CINAHL) were searched from inception to May 4, 2020, using a systematic Boolean search strategy (Additional File 1) developed by an author (U.S.K.). The search was developed without language limits, and it covered articles on or about anesthesia and critical care practice in Africa. Articles in languages none of the authors understood were sent to a professional medical translator on ProZ (http://www.proz.com, ProZ, Syracuse, New York, U.S.A.). After the database search, Google Scholar, ResearchGate, and ORCiD were searched. This hand search was informed using forward and backward citation analyses, i.e., the articles cited by the studies found during the database search and the articles that cited the studies found during the database search.

### Screening

The citations were exported then uploaded on the free online review platform - Rayyan (https://rayyan.qcri.org/, Doha, Qatar). First, duplicates were excluded, then each article was (title and abstract) screened by at least two reviewers (J.T.N., AM, C.W.S., L.D., TA, A.D.N., and D.C.E.). Next, conflicts were resolved by the authors concerned, and if the two authors could not agree, a third author (U.S.K.) was sought for arbitration.

### Data extraction and analysis

Metadata of all the articles included were extracted - article title, year of publication, author affiliations, study design. The data were stored on Google Forms (Google, Menlo Park, CA, U.S.A.) then imported into SPSS v26 (I.B.M., Armonk, NY, U.S.A.). The publication trends, author contributions, and hierarchy of evidence frequencies were calculated and visualized using Tableau Public (Salesforce, Mountain View, CA, U.S.A.).

The absolute number of published articles is a good measure of academic proficiency and research aptitude; however, it does not factor individual contributions at the level of a nation. Countries with more physician anesthesia providers (P.A.P.s) are expected to have higher research outputs. To account for this, the authors chose to calculate the number of articles per African P.A.P.s. This metric was calculated by dividing the number of first author articles by the number of P.A.P.s from each African country. A major barrier to A.C.C.M. research is the lack of protected research time [13]. The P.A.P. density (P.A.P.s per 100,000 population) was used to account for the clinical workload’s effect on research output.

The data on the P.A.P.s (total P.A.P.s and P.A.P.s per 100,000 population) was obtained from a 2015/2016 survey of the World Federation of Societies Anaesthesiologists [14]. Also, correlations were computed between the number of articles and the P.A.P. data. The P.A.P. data of six countries (Botswana, Djibouti, Gambia, Lesotho, Seychelles, and Sudan) was not available, so they were excluded from the analysis. The bivariate analyses (Kruskal Wallis test and Spearman’s correlation) were run and considered statistically significant when the *P*-value < 0.05.

## Results

**Fig. 1:**
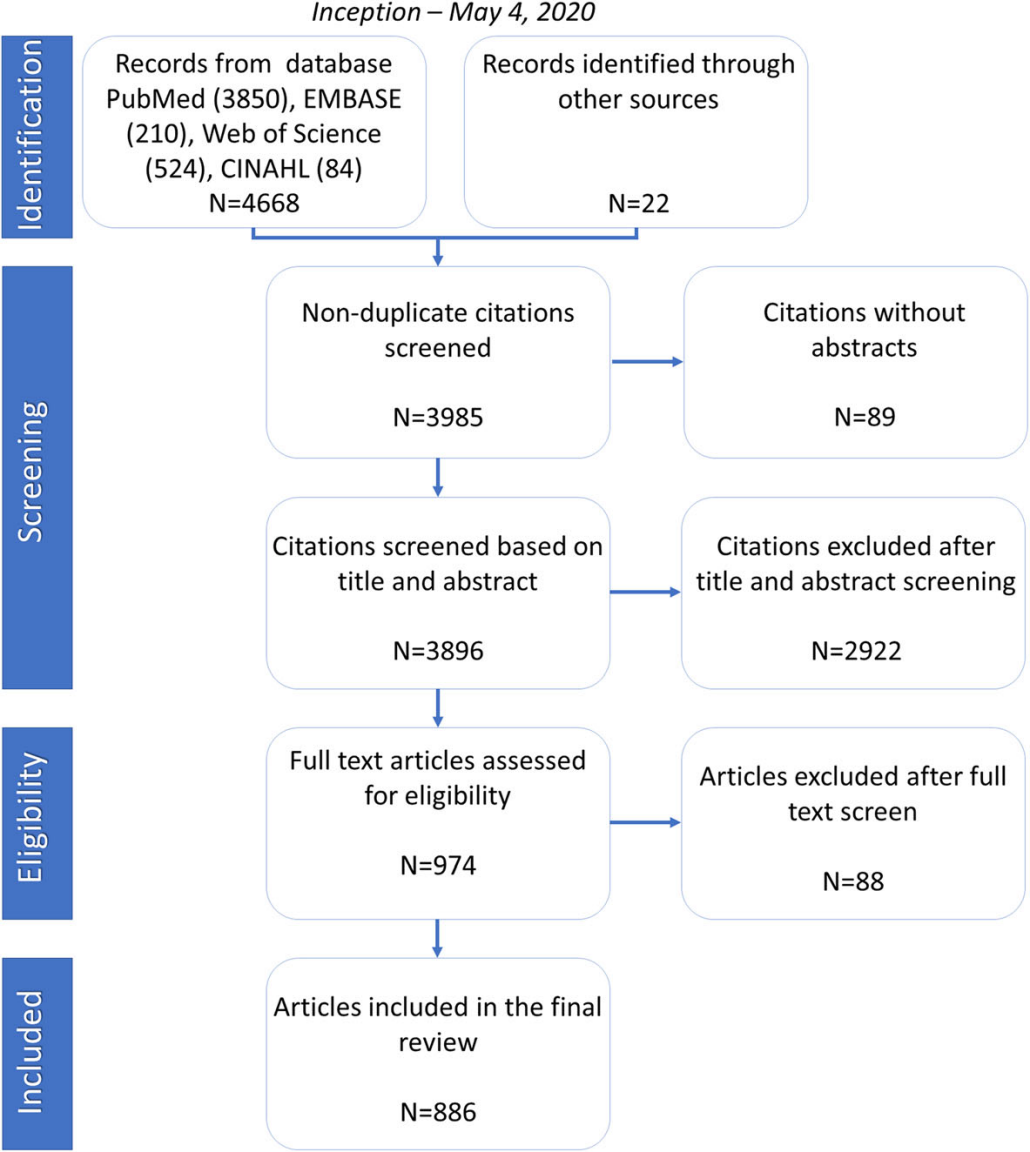
Flow diagram illustrating the search strategy

The search strategy returned 4690 articles: 4688 from databases and 22 from the supplementary hand search. We excluded 705 duplicates and reviewed 3985 non-duplicate articles. After the title and abstract screening, we excluded 2922 more articles because they were irrelevant. Most irrelevant articles returned articles from Papua New Guinea and animal research on guinea pigs in non-African countries (Fig. 1).

### Publication trends and target journals

**Fig. 2:**
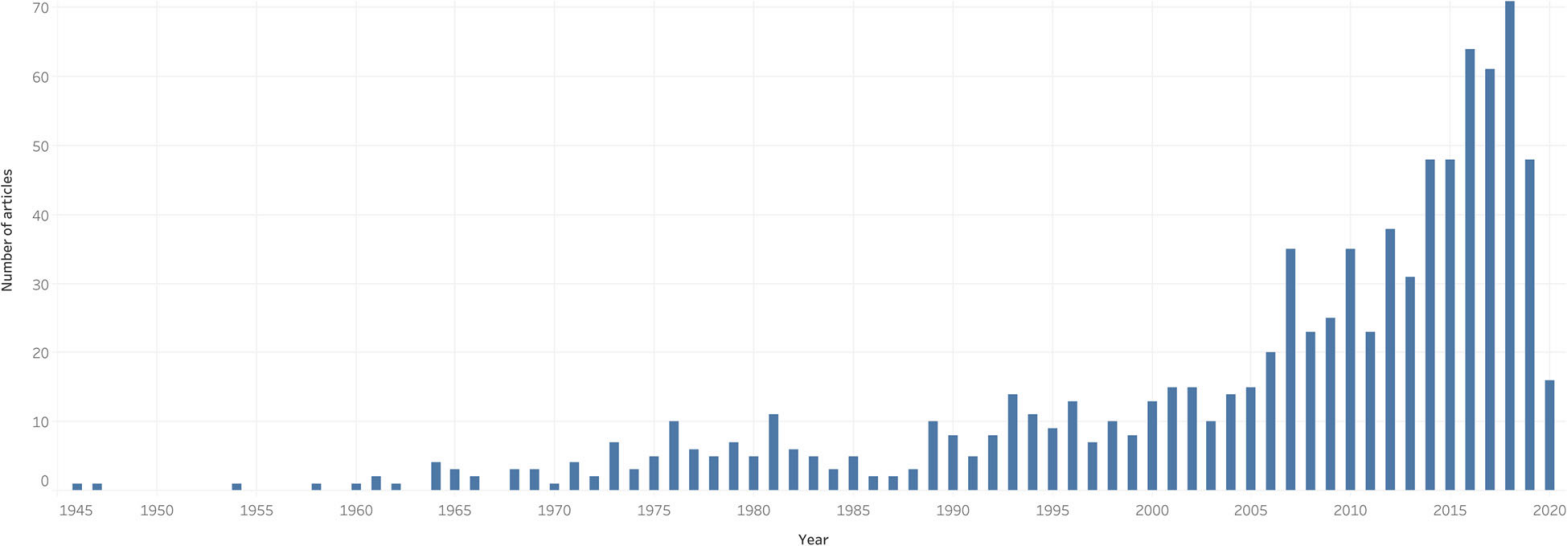
Publication trends in African anesthesia

The final 886 citations were published between 1946 and 2020. The publication trend had three primary cycles with peaks in 1981 (11 articles), 1993 (14 articles), and 2018 (71 articles) (*P* = 0.48) (Fig. 2).

The articles were published in 278 journals. South African Journal of Surgery (55 articles, 6.2%), Anesthesia and Analgesia (51, 5.8%), and Anaesthesia (46, 5.2%) were the top contributors. The top 50 journals are displayed in Table 1.

**Table 1:** Top 50 target journals for African anesthesia research

Journal	Frequency (***N*** = 886)	Percentage (%)
1. South African Journal of Surgery	55	6.2
2. Anesthesia and Analgesia	51	5.8
3. Anaesthesia	46	5.2
4. East African Medical Journal	33	3.7
5. West African Medical Journal	31	3.5
6. Pan African Medical Journal	26	2.9
7. Tropical Doctor	26	2.9
8. Annales Francaises d’Anesthesie et de Réanimation	23	2.6
9. Canadian Journal of Anaesthesia	21	2.4
10. World Journal of Surgery	21	2.4
11. British Journal of Anaesthesia	16	1.8
12. Médecine Tropicale	16	1.8
13. African Journal of Medicine and Medical Sciences	15	1.7
14. International Journal of Gynaecology and Obstetrics	13	1.5
15. The Nigerian Postgraduate Medical Journal	13	1.5
16. African Health Sciences	12	1.4
17. PLoS One	11	1.2
18. La Tunisie Médicale	10	1.1
19. Nigerian Journal of Medicine	10	1.1
20. International Journal of Obstetric Anesthesia	8	0.9
21. Middle East Journal of Anaesthesiology	8	0.9
22. South African Medical Journal	8	0.9
23. Lancet	7	0.8
24. Nigerian Journal of Clinical Practice	7	0.8
25. Dakar Medical	6	0.7
26. Paediatric Anaesthesia	6	0.7
27. African Journal of Paediatric Surgery	5	0.6
28. Anesthesiology	5	0.6
29. B.M.C. Anesthesiology	5	0.6
30. Journal of Obstetrics and Gynaecology	5	0.6
31. Médecine et Santé Tropicales	5	0.6
32. Nigerian Quarterly Journal of Hospital Medicine	5	0.6
33. Anesthesiology Research and Practice	4	0.5
34. B.M.C. Health Services Research	4	0.5
35. B.M.C. Research Notes	4	0.5
36. British Journal of Nursing	4	0.5
37. Journal Français d’Ophtalmologie	4	0.5
38. Journal of Clinical Anesthesia	4	0.5
39. South African Dental Journal	4	0.5
40. Southern African Journal of Anaesthesia and Analgesia	4	0.5
41. The Central African Journal of Medicine	4	0.5
42. Trials	4	0.5
43. African Journal of Reproductive Health	3	0.3
44. Anaesthesia and Intensive Care	3	0.3
45. Annals of The Royal College of Surgeons of England	3	0.3
46. Cahiers d’Anesthésiologie	3	0.3
47. Central African Journal of Medicine	3	0.3
48. Ethiopian Journal of Health Sciences	3	0.3
49. Ethiopian Medical Journal	3	0.3
50. International Anesthesiology Clinics	3	0.3

### Hierarchy of evidence

Most (291, 32.8%) studies had a cross-sectional design. The next most common study designs were cohort studies (172, 19.4%) and randomized controlled trials (76, 8.6%). There were a few animal studies (2, 0.2%) and published guidelines (1, 0.1%) (Additional File 2 Table).

### First author academic output

**Fig. 3:**
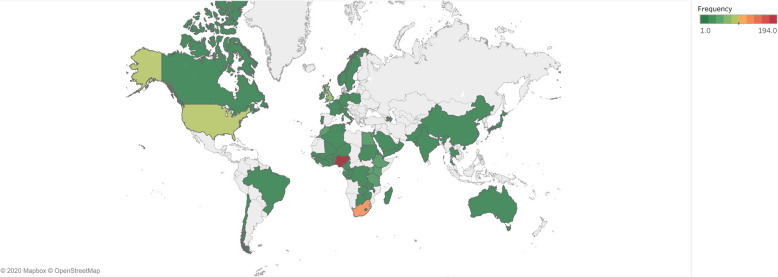
Heat map showing the affiliations of first authors. This map is not under copyright and is freely available to use

The first authors were affiliated with institutions of 62 countries. Most (36, 58.1%) countries were African, and the median number of first author articles by Africans was 8.5 (IQR = 16.25). The most productive first authors were affiliated with institutions from Nigeria (195, 22.0%), South Africa (118, 13.3%), U.S.A. (88, 9.9%), UK (65, 7.3%), and Ethiopia (29, 3.3%) (Fig. 3).

The median number of articles per African P.A.P.s was 0.13 (IQR = 0.38), while the median number of articles per African P.A.P. density was 30.65 (IQR = 88.29) articles. Malawi had the highest number of articles per African P.A.P.s (1.67), followed by Togo (1.06), and Sierra Leone (1.00). When taking into account the P.A.P. density, Ethiopia (580.00), Nigeria (336.21), and Malawi (333.33) were the most significant contributors (Table 2). The number of articles was correlated significantly with the number of African P.A.P.s (*R* = 0.40, *P* = 0.03) but not with the African P.A.P. density (*R* = 0.23, *P* = 0.22).

**Table 2:** Scholarly output per physician anesthesia provider and physician anesthesia provider density

Country	Articles per physician anesthesia provider	Articles per physician anesthesia provider density
Algeria	0.00	0.26
Benin	0.88	93.33
Botswana	NA	NA
Burkina Faso	0.04	7.14
Cameroon	0.42	100.00
Congo, Rep.	0.22	10.53
Côte d’Ivoire	0.04	9.46
Djibouti	NA	NA
D.R.C.	0.05	38.50
Egypt	0.00	3.99
Eritrea	NAN	NAN
Ethiopia	0.57	580.00
Gabon	0.05	0.78
Gambia	NA	NA
Ghana	0.13	35.00
Guinea	0.67	100.00
Kenya	0.12	56.82
Lesotho	NA	NA
Madagascar	0.13	30.43
Malawi	1.67	333.33
Mali	0.02	3.33
Morocco	0.04	14.29
Niger	0.07	14.29
Nigeria	0.19	336.21
Rwanda	0.33	38.24
Senegal	0.20	30.65
Seychelles	NA	NA
Sierra Leone	1.00	66.67
South Africa	0.01	7.29
Sudan	NA	NA
Tanzania	0.42	233.33
Togo	1.06	78.26
Tunisia	0.03	0.04
Uganda	0.31	122.22
Zambia	0.13	20.41
Zimbabwe	0.09	13.25

*NA* Data not available, *NAN* Not a number value

### Second and senior author contributions

There were 245 (27.5%) single-author articles. Similarly to the first authorship, Nigeria was a major contributor among second authors (133, 15.0%). South Africa (77, 8.7%) and the U.S.A. (72, 8.1%) were the second and third major contributors to second author positions.

**Fig. 4:**
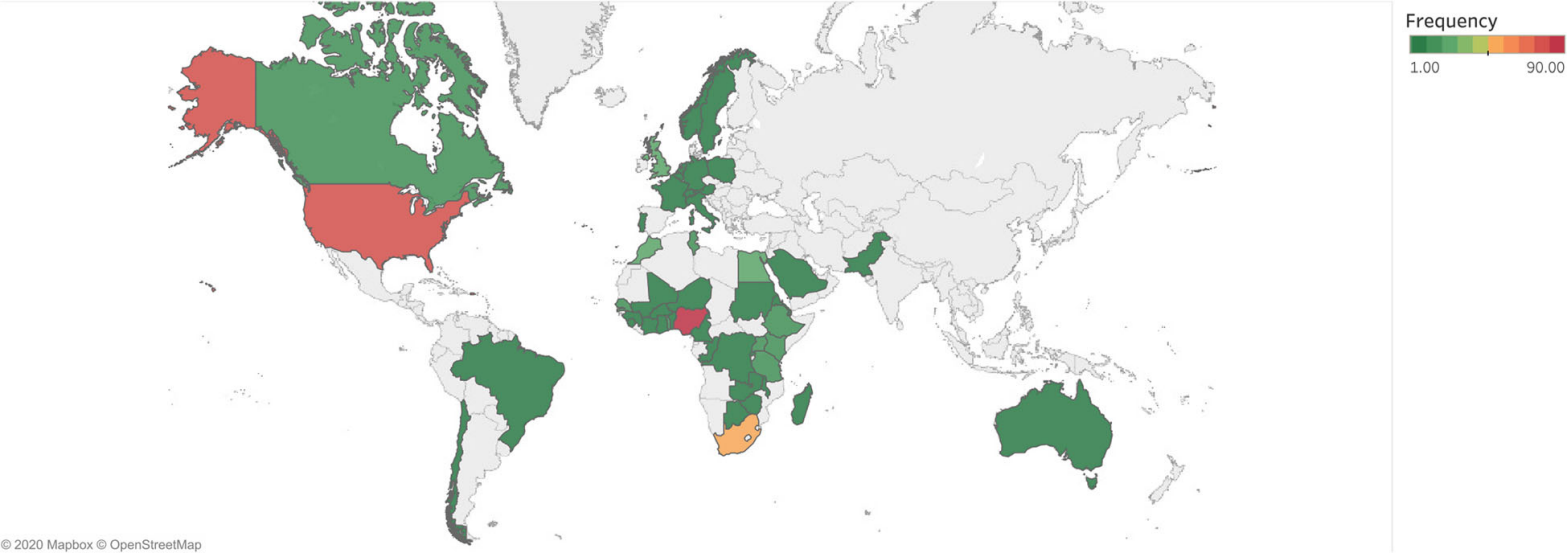
Heat map showing the affiliations of the last authors. This map is not under copyright and is freely available to use

While Nigeria remained the major contributor among first authors (89, 10.0%), South Africa dropped to the third place (47, 5.3%) behind the U.S.A. 74 (8.4%) (Fig. 4).

## Discussion

This study is the first comprehensive analysis of A.C.C.M. research in Africa. African A.C.C.M. publication had cycles of increasing amplitude organized around three peaks. Also, we noted an essential increase in the scholarly output over the last 20 years. The articles were published in a variety of journals, and most studies had an observational study design. Moreover, researchers from Nigeria and South Africa contributed the most to the scholarly output.

### Academic output

Malawi, Sierra Leone, and Togo had the highest number of articles per P.A.P. Malawi ranked among the highest contributors in publications, publications per P.A.P.s, and publications per P.A.P. density. This indicates that Malawian P.A.P.s are proficient researchers despite a higher workload. Both Sierra Leone and Togo have median articles per P.A.P. density below the African median. Moreover, Sierra Leone was adversely affected by the Ebola virus disease and benefited from international aid [15]. This aid fostered the creation of partnerships between local PAPS and foreign P.A.P.s. On the other hand, Togo has trained local P.A.P.s and P.A.P.s from neighboring French-speaking countries, thereby increasing the quantity of Anesthesia research and researchers [16]. Furthermore, Togo and Sierra Leone have relatively small populations (less than 9 million) than other African countries. Hence, increases in the absolute number of articles and P.A.P.s lead to more remarkable changes in the number of articles per P.A.P.s and P.A.P. density.

One-third (18, 33.3%) of African countries did not have first-author publications. This finding is not surprising because African researchers face numerous barriers to publication.

Barriers faced by African A.C.C.M. researchers include lack of funding, institutional support, inexperience, and lack of mentorship [13]. Researchers lack funding to conduct research and to pay for the time spent away from clinical duties. Fortunately, most high impact open access journals offer a sizable discount to researchers from low- and middle-income countries.

African countries, without first author publications, should collaborate with higher-performing countries. The more experienced African researchers can mentor and build capacity among less experienced researchers in neighboring countries. The African Perioperative Research Collaborative is an excellent example of the potential of inter-African A.C.C.M. research collaboratives. The African Perioperative Research Collaborative is a group led by members of the South African Perioperative Research Collaborative that has facilitated A.C.C.M. clinical research among African researchers via technical support, mentorship, and capacity building [4, 17].

A sizable proportion (25, 40.3%) of the first authors were affiliated to non-African institutions, most of them from high-income countries. This might explain why some countries did not have first-author publications. African researchers often assume the role of middle authors when they collaborate with researchers from high-income countries [18]. The lack of first author publications could equally be due to publications in journals that are not indexed in the major databases. Young African researchers often target foreign journals to increase their articles’ visibility because most local journals have low impact factors and small readerships [19–22]. However, African researchers face numerous barriers to publish in international journals, especially if they have high impact factors, and some researchers eventually resort to publishing in predatory journals [22]. These predatory journals do not offer transparent and rigorous peer-review and do not meet the indexation criteria in major databases [23]. Further research is needed to understand the causes of the non-representation of the 18 countries.

The most substantial increase in publications occurred during the early 2000s, and they can be explained by increased interest in Global Anesthesia. Global Anesthesia is a field at the crossroads between anesthesia and public health that focuses on access to safe, timely, and affordable anesthesia care in the world. The World Federation of Societies of Anesthesiologists (W.F.S.A.) and prominent specialty journals supported research on access to and safety in anesthesia care. For example, the W.F.S.A. was a founding member and a sponsor of LifeBox and the World Health Organization Safe Surgery checklist, respectively [3, 5, 7]. Moreover, the W.F.S.A., high-income country academic centers, and non-governmental organizations have developed global anesthesia fellowships in South Africa, Ethiopia, Kenya, and Tanzania, contributing significantly to A.C.C.M. research in Africa. This is evidenced by the sizeable contribution of authors affiliated with non-African institutions to African A.C.C.M. research.

### Hierarchy of evidence

There were few systematic reviews and guidelines among the African A.C.C.M. articles. These two forms of information synthesis are among the highest forms of scientific evidence and should inform practice [24]. The integration of evidence-based medicine to A.C.C.M. is common practice today [25]. However, the implementation of international guidelines to A.C.C.M. practice in Africa is faced with numerous challenges that require adaptation [26, 27]. The adaptation of international guidelines is necessary because the guidelines are based on evidence generated in resource-rich milieus, and there is a dearth of evidence-based and context-specific African guidelines [28, 29]. African researchers must develop these context-specific African guidelines from high-quality scientific evidence. To increase the quality of the evidence generated, African researchers must hone existing skills and acquire new ones. In systematic reviews, researchers can train using the Cochrane Interactive Learning online resource that offers free access to most African nationals [30].

Also, there were few animal studies among the African A.C.C.M. publications. Although animal studies generate lower-grade scientific evidence, they are essential to the development of A.C.C.M.. For example, animal research played an essential role in developing the Guedel cannula, curarisation, and motor blocks [31]. Africa’s rich biodiversity and ethnopharmacology are likely to house the next essential drug in A.C.C.M [32]. This untapped potential could lead to the discovery and production of more affordable drugs for African patients. Unfortunately, African basic science researchers face more barriers than clinical researchers. Researchers are challenged by lack of funding, difficulties obtaining ethical approvals, and inadequate infrastructure [33, 34]. Balogun et al. have proposed the engagement of local and international funding agencies by researchers, the use of invertebrates, and innovative low-cost research methods [35].

### Journals

A significant proportion of the top 50 journals were specialty journals, and only one of the specialty journals was African. Unsustainable financial models can explain the dearth of African specialty journals, lack of editorial expertise, and low submissions quality [36]. All these factors precipitate the failure of young journals. Young specialty journals can curtail the effects of these challenges if they collaborate with more experienced journals. The African Journal Partnership Program provides mentorship and capacity from leading journals of high-income countries to African journals’ editorial teams [37].

The African Journal Partnership Program model should be supplemented by inter-African partnerships between more and less experienced journals. The top contributing journal was South African, and the only African specialty journal was equally South African and emphasized the critical role of South African journals in A.C.C.M. research. The prominence of South African journals is an opportunity for other journals to learn from a successful African editorial staff.

A considerable number of articles were published in non-local high impact factor specialty journals. Similar trends have been observed in Europe and South Asia [38, 39]. Publication in a high impact journal does not guarantee visibility or recognition [40]. Citation metrics are better measures of visibility and recognition [41]; however, we did not collect data on citations. Future studies should evaluate the quantitative and qualitative impact of African A.C.C.M. research. Notwithstanding, the publication of African articles in high impact factor journals attests to the quality of research emanating from the continent.

### The future of A.C.C.M. research in Africa

Going forward, African A.C.C.M. research must adopt a “no woman/man left behind” approach. Continental-level professional groups like the Africa Regional Section of the World Federation of Societies of Anesthesiologists should oversee a continental research agenda focused on capacity-building, especially in countries without first author publications. The capacity-building could be organized online and in-person (concomitantly with continental meetings). These training sessions should be opened not only to specialist physicians but equally to nurse anesthetists, residents, and medical students. Nurse anesthetists, residents, and medical students can help decrease the research workload by contributing to data curation, project administration, and writing of original manuscript drafts. In the absence of protected research time, this strategy can “buy” some time for specialist physicians and build capacity among non-physicians.

### Limitations

We acknowledge the following limitations in our study: our definition of African research excluded Africans’ research about A.C.C.M. in other continents. Next, we considered studies to be equal irrespective of their citation metrics or study design. Therefore, we did not factor the impact of the studies into the contributions. Hence, a letter to the editor with lower citation metrics was considered equal to a systematic review with higher citation metrics. Finally, we limited our analysis to prominent author positions. As such, we failed to capture a detailed picture of the contributors to African A.C.C.M. research. In addition, our search strategy could have been more explicit. We initially opted for a more comprehensive search strategy, but the results returned many irrelevant results. Given our limited resources, we opted for a less broad search. Despite these limitations, we believe our study adds value to existing research.

## Conclusion

This study analyzed the publication trends, study designs, target journals, and contributions to A.C.C.M. research in Africa. There has been an increase in article publication over the past two decades, and the greatest contributors are Nigeria and Malawi. This analysis helped to identify less productive countries, subspecialties, and study designs. As such, our findings can be used to set the A.C.C.M. research agenda in Africa.

## Supplementary information

**Additional file 1.** Search Strategy.

**Additional file 2.** African anesthesia and critical care medicine research output by study design.

## Data Availability

The datasets used and/or analysed during the current study are available from the corresponding author on reasonable request.

## Abbreviations

A.C.C.M: Anesthesia and critical care medicine
P.A.P.s: Physician anesthesia providers
