# Supplementary material for: Systematic review and bibliometric analysis of African anesthesia and critical care medicine research part I: hierarchy of evidence and scholarly productivity
Source: BMC Anesthesiol. 2020 Sep 28;20:247. doi: 10.1186/s12871-020-01167-8 (PMC7523301; doi:10.1186/s12871-020-01167-8)
Supplement: Supplementary file 1 — Additional file 1. Search Strategy. [file 12871_2020_1167_MOESM1_ESM.docx]

**Additional File 1**

**Search strategy**

1. PubMed
   1. (("Anesthesia"[Mesh]OR periopera*[tiab] OR preopera*[tiab] OR postopera* or “critical care”[tiab] OR ressuscita*[tiab])) AND ((("Africa"[Mesh] OR “subsaharan Africa”[tiab] OR “Sub-Saharan Africa”[tiab] OR Algeria[MeSh] OR Angola[MeSh] OR Benin[MeSh] OR Botswana[MeSh] OR Burkina Faso[MeSh] OR Burundi[MeSh] OR Cabo Verde[MeSh] OR Cameroon[MeSh] OR Central African Republic[MeSh] OR Chad[MeSh] OR Comoros[MeSh] OR Congo[MeSh] OR Cote d'Ivoire[MeSh] OR Democratic Republic of the Congo[MeSh] OR Djibouti[MeSh] OR Egypt[MeSh] OR Equatorial Guinea[MeSh] OR Eritrea[MeSh] OR Eswatini[MeSh] OR Ethiopia[MeSh] OR Gabon[MeSh] OR Gambia[MeSh] OR Ghana[MeSh] OR Guinea[MeSh] OR Guinea-Bissau[MeSh] OR Kenya[MeSh] OR Lesotho[MeSh] OR Liberia[MeSh] OR Libya[MeSh] OR Madagascar[MeSh] OR Malawi[MeSh] OR Mali[MeSh] OR Mauritania[MeSh] OR Mauritius[MeSh] OR Morocco[MeSh] OR Mozambique[MeSh] OR Namibia[MeSh] OR Niger[MeSh] OR Nigeria[MeSh] OR Rwanda[MeSh] OR Sao Tome and Principe[MeSh] OR Senegal[MeSh] OR Seychelles[MeSh] OR Sierra Leone[MeSh] OR Somalia[MeSh] OR South Africa[MeSh] OR South Sudan[MeSh] OR Sudan[MeSh] OR Tanzania[MeSh] OR Togo[MeSh] OR Tunisia[MeSh] OR Uganda[MeSh] OR Zambia[MeSh] OR Zimbabwe[MeSh] OR Maghreb*[tiab] OR Benin[tiab] OR Dahomey[tiab] OR Burkina Faso[tiab] OR Upper Volta[tiab] OR Burundi[tiab] OR Urundi[tiab] OR Central African Republic[tiab] OR Ubangi-Shari[tiab] OR Chad[tiab] OR Comoros[tiab] OR Mayotte[tiab] OR “Democratic Republic of Congo”[tiab] OR “Democratic Republic of the Congo”[tiab] OR “Congo, Dem. Rep”[tiab] OR Kinshasa[tiab] OR “Belgian Congo”[tiab] OR Zaire[tiab] OR Katanga[tiab] OR Eritrea[tiab] OR Ethiopia[tiab] OR Gambia[tiab] OR Guinea-Bissau[tiab] OR Guinea[tiab] OR Liberia[tiab] OR Madagascar[tiab] OR “Malagasy Republic”[tiab] OR Malawi[tiab] OR Nyasaland[tiab] OR Mali[tiab] OR Mozambique[tiab] OR “Portuguese East Africa”[tiab] OR OR Niger[tiab] OR Rwanda[tiab] OR Ruanda[tiab] OR Sao Tome and Principe[tiab] OR Senegal[tiab] OR Sierra Leone[tiab] OR Somalia[tiab] OR South Sudan[tiab] OR Tanzania[tiab] OR Zanzibar[tiab] OR Tanganyika[tiab] OR Togo[tiab] OR Togolese Republic[tiab] OR Uganda[tiab] OR Zambia[tiab] OR Zimbabwe[tiab] OR Southern Rhodesia[tiab]))) NOT “guinea pig”[tiab]
   2. Date of search: May 4, 2020
   3. Results: 3850 citations
2. Embase
   1. Search strategy
      ('africa'/exp OR 'low income country'/exp OR 'subsaharan africa':ab,ti OR 'sub-saharan africa':ab,ti OR 'maghreb*':ab,ti OR ssa:ab,ti OR 'benin'/exp OR 'burkina faso'/exp OR 'burundi'/exp OR 'central african republic'/exp OR 'chad'/exp OR 'comoros'/exp OR 'democratic republic congo'/exp OR 'eritrea'/exp OR 'ethiopia'/exp OR 'gambia'/exp OR 'guinea bissau'/exp OR 'guinea'/exp OR 'liberia'/exp OR 'madagascar'/exp OR 'malawi'/exp OR 'mali'/exp OR 'mozambique'/exp OR 'niger'/exp OR 'rwanda'/exp OR 'senegal'/exp OR 'sierra leone'/exp OR 'somalia'/exp OR 'south sudan'/exp OR 'tanzania'/exp OR 'togo'/exp OR 'uganda'/exp OR 'zambia'/exp OR 'zimbabwe'/exp OR benin:ab,ti OR dahomey:ab,ti OR 'burkina faso':ab,ti OR 'upper volta':ab,ti OR burundi:ab,ti OR urundi:ab,ti OR 'central african republic':ab,ti OR 'ubangi shari':ab,ti OR chad:ab,ti OR comoros:ab,ti OR mayotte:ab,ti OR 'republic of congo':ab,ti OR 'democratic republic of congo':ab,ti OR brazzaville:ab,ti OR 'french congo':ab,ti OR 'democratic republic of the congo':ab,ti OR 'congo, dem. rep':ab,ti OR kinshasa:ab,ti OR 'belgian congo':ab,ti OR zaire:ab,ti OR katanga:ab,ti OR eritrea:ab,ti OR ethiopia:ab,ti OR gambia:ab,ti OR 'guinea bissau':ab,ti OR guinea:ab,ti OR haiti:ab,ti OR liberia:ab,ti OR madagascar:ab,ti OR 'malagasy republic':ab,ti OR malawi:ab,ti OR nyasaland:ab,ti OR mali:ab,ti OR mozambique:ab,ti OR 'portuguese east africa':ab,ti OR niger:ab,ti OR rwanda:ab,ti OR ruanda:ab,ti OR senegal:ab,ti OR 'sierra leone':ab,ti OR somalia:ab,ti OR 'south sudan':ab,ti tanzania:ab,ti OR zanzibar:ab,ti OR tanganyika:ab,ti OR togo:ab,ti OR 'togolese republic':ab,ti OR uganda:ab,ti OR zambia:ab,ti OR zimbabwe:ab,ti OR 'southern rhodesia':ab,ti) AND ('anesthesia'/exp)
   2. Date of search: May 4, 2020
   3. Results: 210
3. Web of science
   1. Search strategy
      TS=(anesthe* AND africa) OR TI=((Anesthe* OR periopera* OR postopera* or preopera*) AND ("Africa" OR "subsaharan Africa" OR "Sub-Saharan Africa" OR Algeria OR Angola OR Benin OR Botswana OR Burkina Faso OR Burundi OR Cabo Verde OR Cameroon OR Central African Republic OR Chad OR Comoros OR Congo OR Cote d'Ivoire OR Democratic Republic of the Congo OR Djibouti OR Egypt OR Equatorial Guinea OR Eritrea OR Eswatini OR Ethiopia OR Gabon OR Gambia OR Ghana OR Guinea OR Guinea-Bissau OR Kenya OR Lesotho OR Liberia OR Libya OR Madagascar OR Malawi OR Mali OR Mauritania OR Mauritius OR Morocco OR Mozambique OR Namibia OR Niger OR Nigeria OR Rwanda OR Sao Tome and Principe OR Senegal OR Seychelles OR Sierra Leone OR Somalia OR South Africa OR South Sudan OR Sudan OR Tanzania OR Togo OR Tunisia OR Uganda OR Zambia OR Zimbabwe OR Maghreb* OR Benin OR Dahomey OR Burkina Faso OR Upper Volta OR Burundi OR Urundi OR Central African Republic OR Ubangi-Shari OR Chad OR Comoros OR Mayotte OR "Democratic Republic of Congo" OR "Democratic Republic of the Congo" OR "Congo, Dem. Rep" OR Kinshasa OR "Belgian Congo" OR Zaire OR Katanga OR Eritrea OR Ethiopia OR Gambia OR "Guinea-Bissau" OR Guinea OR Liberia OR Madagascar OR "Malagasy Republic" OR Malawi OR Nyasaland OR Mali OR Mozambique OR "Portuguese East Africa" OR Niger OR Rwanda OR Ruanda OR "Sao Tome and Principe" OR Senegal OR Sierra Leone OR Somalia OR South Sudan OR Tanzania OR Zanzibar OR Tanganyika OR Togo OR "Togolese Republic" OR Uganda OR Zambia OR Zimbabwe OR "Southern Rhodesia")) NOT TI=("guinea pig*" OR animal)
   2. Date of search: May 4, 2020
   3. Results: 524
4. CINAHL
   1. Search strategy:
      Title or abstract: "Africa" OR "subsaharan Africa" OR "Sub-Saharan Africa" OR Algeria OR Angola OR Benin OR Botswana OR Burkina Faso OR Burundi OR Cabo Verde OR Cameroon OR Central African Republic OR Chad OR Comoros OR Congo OR Cote d'Ivoire OR Democratic Republic of the Congo OR Djibouti OR Egypt OR Equatorial Guinea OR Eritrea OR Eswatini OR Ethiopia OR Gabon OR Gambia OR Ghana OR Guinea OR Guinea-Bissau OR Kenya OR Lesotho OR Liberia OR Libya OR Madagascar OR Malawi OR Mali OR Mauritania OR Mauritius OR Morocco OR Mozambique OR Namibia OR Niger OR Nigeria OR Rwanda OR Sao Tome and Principe OR Senegal OR Seychelles OR Sierra Leone OR Somalia OR South Africa OR South Sudan OR Sudan OR Tanzania OR Togo OR Tunisia OR Uganda OR Zambia OR Zimbabwe OR Maghreb* OR Benin OR Dahomey OR Burkina Faso OR Upper Volta OR Burundi OR Urundi OR Central African Republic OR Ubangi-Shari OR Chad OR Comoros OR Mayotte OR "Democratic Republic of Congo" OR "Democratic Republic of the Congo" OR "Congo, Dem. Rep" OR Kinshasa OR "Belgian Congo" OR Zaire OR Katanga OR Eritrea OR Ethiopia OR Gambia OR "Guinea-Bissau" OR Guinea OR Liberia OR Madagascar OR "Malagasy Republic" OR Malawi OR Nyasaland OR Mali OR Mozambique OR "Portuguese East Africa" OR Niger OR Rwanda OR Ruanda OR "Sao Tome and Principe" OR Senegal OR Sierra Leone OR Somalia OR South Sudan OR Tanzania OR Zanzibar OR Tanganyika OR Togo OR "Togolese Republic" OR Uganda OR Zambia OR Zimbabwe OR "Southern Rhodesia" AND
      Title or abstract: Anesthe* OR periopera* OR postopera* or preopera*
   2. Date of search: May 4, 2020
   3. Results: 84
5. Supplementary hand search: **African journals online**, references of articles included, contact experts in the field to make sure nothing has been missed, Google scholar, theses websites
   1. Search strategy
   2. Date of search
   3. Results
