# Supplementary material for: Systematic review and bibliometric analysis of African anesthesia and critical care medicine research part I: hierarchy of evidence and scholarly productivity
Source: BMC Anesthesiol. 2020 Sep 28;20:247. doi: 10.1186/s12871-020-01167-8 (PMC7523301; doi:10.1186/s12871-020-01167-8)
Supplement: Supplementary file 2 — Additional file 2. African anesthesia and critical care medicine research output by study design. [file 12871_2020_1167_MOESM2_ESM.docx]

**Additional File 2**

**Table**

**African anesthesia and critical care medicine research output by study design**

| **First author affiliation** | **Randomized controlled studies, systematic reviews and meta-analyses** | **Observational and other non-randomized studies** | **Animal studies** | **Letters, commentaries, editorials** | **Unavailable** |
| --- | --- | --- | --- | --- | --- |
| Algeria  Australia  Azerbaijan  Belgium  Benin  Botswana  Brazil  Burkina Faso  Cameroon  Canada  Chile  China  Congo, Rep.  Côte d'Ivoire  Djibouti  DRC  Egypt  Eritrea  Ethiopia  France  Gabon  Gambia  Germany  Ghana  Guinea  India  Ireland  Israel  Italy  Japan  Kenya  Lesotho  Madagascar  Malawi  Mali  Morocco  Niger  Nigeria  Norway  Oman  Pakistan  Poland  Portugal  Rwanda  Saudi Arabia  Senegal  Seychelles  Sierra Leone  South Africa  Sudan  Sweden  Switzerland  Tanzania  Thailand  Togo  Tunisia  Uganda  UK  USA  Yemen  Zambia  Zimbabwe | 0  1  0  0  3  0  0  0  1  4  1  0  0  0  0  1  15  0  0  1  0  0  0  1  0  0  0  0  0  0  6  0  0  1  0  1  1  20  0  0  1  0  0  1  1  0  0  0  5  0  1  1  1  1  0  3  3  5  12  0  0  0 | 1  4  1  2  9  2  1  2  6  8  0  1  2  3  2  3  6  1  28  9  0  1  9  13  2  0  1  2  3  1  15  0  7  7  1  21  1  141  1  1  0  4  1  6  1  16  1  2  81  6  5  0  17  0  16  10  18  44  59  1  9  6 | 0  0  0  0  0  0  0  0  0  0  0  0  0  0  0  0  0  0  0  0  0  0  0  0  0  0  0  1  0  0  0  0  0  0  0  0  0  0  0  0  0  0  0  0  0  0  0  0  0  0  1  0  0  0  0  0  0  0  0  0  0  0 | 0  2  0  1  1  0  0  0  1  7  0  0  0  0  0  1  0  0  0  1  1  1  2  0  0  1  0  1  0  0  2  1  0  1  0  0  0  2  0  0  0  0  0  2  0  0  0  0  18  0  0  0  1  0  0  1  1  10  12  0  1  2 | 0  1  0  0  1  0  0  0  2  1  0  0  0  4  0  0  3  0  1  0  0  0  1  0  0  0  0  0  1  0  2  0  0  1  0  5  0  32  0  0  0  0  0  4  0  3  0  0  14  0  0  1  2  0  2  3  0  6  5  0  0  3 |
